# Putting People at the Centre of Guidelines

**DOI:** 10.1002/gin2.70054

**Published:** 2025-11-13

**Authors:** Karen Graham, Naomi Fearns

**Affiliations:** ^1^ Scottish Intercollegiate Guidelines Network Edinburgh Great Britain UK; ^2^ Healthcare Improvement Scotland Edinburgh Great Britain UK

## Abstract

- Patient and Public Involvement (PPI) is central: SIGN guidelines are shaped by people with lived experience, improving quality and relevance.
- The article uses the SIGN Dementia guideline as a case study: Carers and third sector organisations helped ensure person‐centred care and influenced the inclusion of topics like anticipatory grief.
- Inclusive engagement methods: SIGN used virtual meetings, presentations, and peer review to gather feedback from people with dementia and carers throughout development.
- Challenges addressed: The team managed power dynamics and communication barriers, ensuring all voices were heard and valued.
- Real impact: Lived experience led to changes in recommendations, such as adding sections on grief and regular care plan reviews.
- Conclusion: Inclusive PPI results in more practical, trusted, and relevant guidelines for all stakeholders.

## 1 Introduction

In our 30 years of producing guidelines, the Scottish Intercollegiate Guidelines Network (SIGN) has consistently adhered to the principles of Appraisal of Guidelines for Research and Evaluation (AGREE II) [1], prioritising Patient and Public Involvement (PPI) as set out in our SIGN 50 methodology handbook [2]. This commitment to PPI helps SIGN to produce high‐quality clinical guidelines, that foster shared decision‐making by recognising the outcomes that are important to people with lived experience, their values and their preferences [3, 4, 5, 6, 7, 8]. Despite its value in guideline development, we recognise that more evaluation and research is needed to provide evidence of the impact of PPI on guideline quality, acceptability, implementation success and health outcomes [9, 10]. SIGN works in partnership with professionals, the third sector (non‐profit organisations and charities), people with lived experience and the public, which enhances our guidelines credibility and helps us to tailor them to meet the real‐world needs of all interest‐holders [5, 6, 7, 8, 11, 12]. In summary, PPI helps to make guideline recommendations better by incorporating patient preferences and values into them, and this helps guideline developers to achieve their aim of enhancing patient care and improving health outcomes [3, 11, 12, 13, 14].

In this commentary we showcase the significance of PPI in the development of clinical guidelines by SIGN, using its dementia guideline [15], as a case example. We provide insights into SIGN's collaborative efforts, reinforcing the importance of diverse perspectives at each stage of the guideline development process.

## 2 Case Example

The SIGN guideline on Assessment, diagnosis, care and support for people with dementia and their carers was published in 2023 [15]. It encourages all health and social care professionals to adopt person‐centred care and treat people living with dementia and unpaid carers as individuals. The methodology we used for developing this guideline is outlined in SIGN 50 [2]. The quotes used to illustrate the commentary are drawn from feedback provided from carers and third sector organisation representatives gathered through surveys conducted during development of the guideline.

Representatives from third sector organisations and carers of people with dementia were members of our guideline development group and worked in partnership with professionals to produce the guideline. Carers played a crucial role in ensuring that issues that they saw as priorities, such as anticipatory grief, were thoroughly considered and addressed.

During the evaluation of their experiences of working with us, a carer provided feedback stating:

I felt strongly, as did many of my National Dementia Carers Action Network fellow carers and former carers, that grief needed to be looked into at various stages, from pre diagnosis, at diagnosis, post diagnosis and during the advancing stages of the illness. I also felt that we introduced the link between guilt and grief experienced by carers, throughout the patient's journey, and after death. This changed the direction of the guideline to include a specific section on the different kinds of grief and how these might be recognised and treated.

(Carer on guideline development group)

Third sector organisations invited people with dementia and their carers to participate in our guideline development processes to ensure diverse perspectives influenced decisions. We held multiple sessions with people with dementia, to ensure their perspectives were integrated into the wording and phrasing of the guideline, enhancing its practical relevance and authenticity. This collaborative approach aimed to ground the guideline in real‐world experiences, as well as evidence, making it more applicable and beneficial to those directly affected by dementia.

During the evaluation of their experiences of working with us, a third sector organisation representative provided feedback stating

From generously sharing their unique, personal experiences of living with dementia or supporting someone living with dementia to offering practical advice and guidance to ensure that the guideline is truly accessible to non‐professionals, our members have been influential in the development of the guideline.

(Representative, Alzheimer's Scotland)

## 3 Methods of Engagement

Engaging with people with lived experience and relevant organisations early in the guideline development process is essential to allow their views and experiences to shape the guideline. Table 1 outlines the range of strategies used to engage people in our dementia guideline. The quote below from a carer on the guideline group recognises the importance of using a variety of PPI methods throughout the guideline development process:

Because there were just two lived experience representatives [on the guideline development group], I am so glad that there was a variety of consultations with people living with dementia and carers throughout various stages of development, and in the final stages before publication.

(Carer on guideline group)

**Table 1:** Inclusive strategies supporting the SIGN dementia guideline.

Strategy	Description
Inviting people with dementia and their carers to a virtual meeting.	We organised a virtual meeting via Microsoft Teams where people with dementia and their carers could share their views, needs and priorities. This insured their voices were heard at the beginning of the process.
Inviting third sector organisations to give a presentation at the first meeting of the guideline group.	We invited third sector organisations to give a presentation at the first guideline group meeting to share the issues they had highlighted during the scoping exercise. This provided valuable insights from organisations experienced in dementia care.
Inviting people with dementia through third sector organisations to a pre‐consultation meeting to discuss plans for the consultation meeting and how we could support their involvement.	We arranged a pre‐consultation meeting via Microsoft teams. This helped us to plan how to make the consultation meeting accessible to people with dementia and to present recommendations in a way that people with dementia would understand.
Inviting people with dementia to take part in the peer review process.	We invited people with dementia to provide feedback on the draft guideline via our peer review process. We responded to their suggestions and changed the guideline based on their feedback.

## 4 Challenges in PPI

Supporting meaningful engagement requires significant preparation and planning to involve the right people. We ensured our processes were inclusive and accommodated a wide range of needs and preferences. As facilitators of guideline group meetings, we needed to understand the specific needs of people with lived experience, ensuring they had adequate time and space to express themselves [14]. When involving people with lived experience in consultation meetings for the dementia guideline, we implemented strategies to include people with cognitive impairments to ensure their voices were heard and valued. This comprehensive approach to PPI not only enriched the decision‐making process but also fostered a more inclusive and empathetic environment. Effective communication, and making reasonable adjustments [2, 12] for people's support needs, allows people with dementia to share their experiences and views. We used techniques, such as clear, simple language during discussions and allowing extra time for responses and repetition. By prioritising these strategies, we ensured that the voices of people with dementia were heard and valued. Further details on the equality considerations underpinning this study can be found in the Equality Impact Assessment report, available on the SIGN website [14].

We directly asked people with dementia what would help them in communicating with us. This empowered them to express their preferences and needs, fostering a more person‐centred approach. We tailored our communication strategies to better suit their individual requirements, enhancing their participation and engagement [16]. We recognised the importance of allowing a carer(s) to accompany the person with dementia to meetings. We found that the carers helped interpret the needs and preferences of the person with dementia and ensured that their concerns were adequately addressed.

Balancing power dynamics within the guideline development group was crucial to support all interest‐holders to be able to contribute. If power imbalances are not addressed, guideline development processes are likely to favour more vocal participants. We tried to include everyone fairly and encourage all voices to be heard. While there were some disagreements between those with lived experience and professionals due to their differing perspectives and knowledge bases, this diversity of thought ultimately enriched the group's collective thinking.

An example of the importance of recognising power dynamics was evident during the research question setting stage of the guideline development process. At a guideline meeting, the chair reminded the group of the value of having a carer on the group and allocated protected time for the carer and third sector representatives to share their views on the questions. As a result, an additional research question was added based on their input. Feedback from group members indicated that this area would have been overlooked if the carer and third sector representatives had not had protected time to express their views.

During the evaluation of their experiences of working with us, a third sector organisation representative provided feedback stating:

Having carers on the guideline [development] group certainly enhanced the guideline and ensured their views were taken into account. There were tensions between those with lived experience and experience from clinical practice as these experiences were based on different knowledge bases. I think involving lived experience in the group may have challenged some individual's thinking which is a positive.

(Representative, About Dementia, Age Scotland)

## 5 Insights From People With Lived Experience

Incorporating insights from people with lived experience at the scoping, research question setting and consultation stages (including feedback on draft guidelines) enhances the relevance and effectiveness of guidelines [8, 17]. Participation as guideline development group members ensures peoples perspectives are considered during development and fosters a sense of ownership and trust in the guideline. Gathering many views from people with lived experience via consultation allows a broader range of experiences and options to be considered, helping to make the guideline more comprehensive and reflective of the community's needs [5, 7, 10, 12, 16, 17, 18]. A notable example of this is seen in the inclusion of a research question on grief management at all stages of dementia due to the input of a carer on the guideline group.

During the evaluation of their experiences of working with us, a carer provided feedback stating:

My main contribution was probably getting professionals to accept my feelings that depression is too often diagnosed when people living with dementia, their unpaid carers and family are all suffering from a form of grief—pre death or anticipatory grief from diagnosis through to death.

(Carer on guideline group)

To better reflect the views and needs of people with dementia, we modified an infographic in the guideline on post‐diagnostic support. These changes ensured that the guideline was not only theoretically sound but also practically applicable and supportive. An important change made following consultation was based on a person with dementia's comment emphasising the importance of regular reviews and anticipatory care plans (ACPs) for people with dementia. Although, not legally required, the guideline was updated to reflect the good practice of regular ACP reviews, demonstrating the positive impact of patient and public involvement.

## 6 Conclusion

SIGN's commitment to patient and public involvement has been instrumental in producing high‐quality person‐centred guidelines. By involving professionals, third sector organisations and people with lived experience, we have created a more inclusive and responsive guideline development process. This approach, particularly evident in our dementia guideline, ensures that guidelines are both evidence‐based and grounded in real‐world experiences.

Integrating patient and public involvement into the SIGN guideline on dementia presented challenges such as balancing diverse perspectives, overcoming communication barriers and managing emotional discussions. These were addressed through inclusive engagement, creating a supportive environment and tailored communication strategies. The benefits of PPI in the dementia guideline included increased stakeholder engagement, enhanced trust in the guideline, and the creation of practical and relevant recommendations for all interest‐holders.

## Author Contributions

**Karen Graham:** writing – original draft, writing – review and editing. **Naomi Fearns:** writing – original draft, writing – review and editing.

## Conflicts of Interest

The authors declare no conflicts of interest.

## Data Availability

Data sharing is not applicable to this article as no new data were created or analysed in this study.
